# Tunnel lining crack expansion and maintenance strategy optimization considering train loads: A case study

**DOI:** 10.1371/journal.pone.0290533

**Published:** 2023-08-25

**Authors:** Dapeng Wang, Jingchun Wang, Chengjie Rao, Xing Niu, Qiang Xu

**Affiliations:** 1 School of Civil Engineering, Shijiazhuang Tiedao University, Shijiazhuang, Hebei, China; 2 School of Safety Engineering and Emergency Management, Shijiazhuang Tiedao University, Shijiazhuang, Hebei, China; Fuzhou University, CHINA

## Abstract

Cracks in concrete tunnel linings are inevitable during service life. It is necessary to keep abreast of the cracking condition of the lining and formulate reasonable inspection and maintenance measures to ensure operational safety. Considering the influence of train loads on the safety and service performance of cracked linings, the expansion process of lining cracks and the maintenance strategy of tunnels during the service period was investigated. The impact of detection probability and maintenance measures on the service life of tunnel lining and the cost of detection and maintenance of cracked lining in the whole life cycle was analyzed; the optimization calculation model of tunnel lining crack detection and maintenance strategy based on genetic algorithm was established with the multi-objective optimization function of maximizing the service life of detection and maintenance and minimizing the total cost of detection and maintenance of fatigue cracks. The optimization analysis of lining crack expansion, detection, and maintenance was carried out for an operational railroad tunnel. Finally, an optimization analysis of lining crack expansion and maintenance was carried out in a railway tunnel. The results show that the stress intensity factor at the tip of the lining cracks is the same as the train load waveform; the magnitude of the stress intensity factor approximately satisfies the exponential function relationship with the depth of cracks; the fatigue service life of cracked lining is positively correlated with the cost of inspection and maintenance; the adoption of the necessary maintenance and the increase in the number of inspections and maintenance have a better economy while meeting the expectation of the service life. According to the Pareto solution set, the management can formulate the inspection and maintenance strategy based on the tunnel’s expected life and maintenance budget.

## 1 Introduction

The tunnels play a crucial role in rail transport as an essential infrastructure. By the end of 2022, China has 17,873 railroad tunnels in operation, with a total length of 21,978 km, in which 4,178 high-speed railroad tunnels with a total length of 7,032 km are in operation [1]. Along with the vigorous development of tunnel engineering, various types of damages generated during the service period also significantly affect the tunnels’ performance [2], and the extreme neglect of these effects may lead to severe consequences. Incidents such as corrosion of sulfate on the North London Line in the UK [3], rusting of subway lining in Hong Kong [4], and ceiling collapse in the Sasago tunnel in Tokyo, Japan [5], it is clear that the deterioration of tunnel structures has caused substantial economic losses and human casualties worldwide [6]. However, current tunnel maintenance methods are mostly reactive due to poor and untimely planning. Attending to problems after they have occurred usually implies higher remediation costs than taking timely action. Reactive maintenance methods may lead to severe consequences that jeopardize the performance and safety of the tunnel due to the aging degradation mechanisms and various loading effects. Thus, the development of reasonable inspection and maintenance measures has been vital to ensure the secure operational maintenance of tunnels.

Secondary lining cracking is one of the earliest signs of tunnel deterioration, which poses a significant maintenance challenge [7]. Modern tunnel mechanics considers the initial support and the surrounding rock as the primary stressed structure, and thus the deterioration due to the external environment may not have a significant effect on the secondary lining cracks. More importantly, it should be noted that train vibration loads have a significant impact on the performance of cracked linings. Gharehdash & Barzegar [8] investigated the effect of moving train loads on tunnel lining using numerical models and found that vibratory loads cause shear damage. Wang et al. [9] proposed a method for analyzing the stability of concrete lining cracks considering two fracture criteria in combination with testing data. Liu et al. [10] investigated tunnel inverted arch fatigue behavior under cyclic loading and pointed out that the fatigue damage is related to the train load amplitude. Liu and Han [11] established the grading criteria for tunnel lining cracking disease by fracture mechanics using crack depth as a parameter. Xu et al. [12] analyzed the cracking characteristics of tunnel secondary lining cracks by model tests based on the results of field investigations. Xu & Ma [13] analyzed the expansion law of lining cracks under the action of train cycles and calculated the stress intensity factors (SIFs) at different crack depths. Du et al. [14] studied the crack propagation law of tunnel invert under the influence of train load based on field tests. The above studies have extensively analyzed the loading patterns and dynamic loading characteristics of cracked linings under train loads, which provide essential references for the safety assessment of cracked linings. Still, these studies on the service performance of cracked linings are mainly concerned with the existing crack state, ignoring the crack expansion caused by train loads during the service period.

Inspection and maintenance are essential instruments to reduce and control the risk of structural failure. Too much testing and maintenance can increase maintenance costs, while less may lead to lower reliability than the target value and leave the structure in an unsafe condition [15]. Therefore, a reasonable inspection and maintenance strategy should balance cost and risk well [16]. Baji et al. [17] proposed a framework for optimizing maintenance policies based on different failure modes by solving the optimal solution between tunnel risk and maintenance cost for the continuous deterioration of tunnel structures under groundwater and soil interaction. Han et al. [18] proposed a performance-based tunnel maintenance strategy given lining deterioration due to carbonation and chloride ion erosion. Ai et al. [19] addressed the tunnel’s different degradation modes, suggesting that developing a non-scheduled inspection policy would be more beneficial to reduce the maintenance cost and risk of tunnels. Petroutsatou et al. [20] presented a life cycle model for estimating road tunnel costs, relying on tunnel data from the Egnatia Odos Motorway in northern Greece. Although well developed, the fundamental problem remains that the evaluations used as input often proceed from the ideal state of the tunnel and fails to take into account initial defects in the lining structure, such as cracks, voids, etc. Studies have shown that many tunnels contain initial defects at the beginning of operation due to improper construction, concrete creep, design, and geological conditions [21–23], and if the effects of initial defects are not considered, then the maintenance strategy developed will be conservative.

This paper presents a framework for estimating crack expansion over the entire lifetime based on train loads, which provides tunnel managers with critical decision-making information for planning secondary lining maintenance activities. A framework calculates the tunnel’s secondary lining structural cracks remediation cost, since the lining cracking is one of the most common issue that requires tunnel manager’s attention. An optimization function with multiple objectives of maximizing the service life after inspection and maintenance and minimizing the total cost of inspection and maintenance is established by analyzing the crack growth process. A multi-objective optimization algorithm model is constructed for tunnel lining whole-life maintenance strategy based on genetic algorithm. A tunnel detection and maintenance strategy is formed for crack expansion due to train loading, using an operational railroad tunnel containing lining cracks as the engineering basis. The results of the study can analyze and quantify the effect of train loads on the service performance of cracked tunnel linings, which not only helps to strengthen the weak points of the lining and effectively improve the durability of tunnels but also provides a reference for the tunnel management to formulate appropriate inspection and maintenance strategies.

## 2 Paris formula for fatigue crack expansion

Fatigue cracks in concrete linings generally proceed through crack formation and expansion [24, 25]. Due to the properties of the concrete structure itself, design, and construction process, it inevitably has initial defects that lead to a fatigue crack formation life approaching zero. Therefore, only their fatigue expansion life under the action of train loads should be considered for considering tunnel linings containing initial cracks.

Various analysis methods for fatigue crack growth have been formed, and the most widely used way in engineering is still the fatigue crack growth formula proposed by Paris and Erdogan [26] in 1963 based on metal material experiments. It establishes the relationship between stress intensity factor and crack growth rate, which is the basis of the theory of predicting fatigue crack growth life in engineering applications today. Baluch et al. [27] and Perdikaris et al. [28] introduced the Paris formula to study the fatigue propagation of concrete cracks. Ortiz et al. [29] and Sobczyk et al. [30] used the Paris formula of parameter randomization to estimate the first overstep of concrete fatigue cracks to a certain threshold. Using the Paris formula, it is of great practical significance to analyze and predict the residual life of concrete structures with defects that still need to be kept in service.

The Paris formula is expressed as follows:

$$\frac{da}{dN}=C{\left(\Delta K\right)}^{m}$$ (1)

Where *C* and *m* describe the basic parameters of the material fatigue crack expansion properties, where *C* is related to the structure size and *m* to the notch-to-depth Ratio, d*a*/d*N* is the crack expansion rate, *a* is the crack depth, *N* is the number of load actions, and Δ*K* is the stress intensity factors (SIFs) amplitude.


$$\Delta K=S_{sr}Y(a)\sqrt{\pi a}$$ (2)

Where *S*_sr_ is the stress amplitude; *Y*(*a*) is the geometric function.

Assuming that the initial crack size of the structure is *a*_0_, according to Eqs (1) and (2), when the crack size expands from *a*_0_ to *a*, the number of load actions can be expressed as:

$$N=\frac{1}{CS_{{}_{sr}}^{m}}\int_{a_{0}}^{a}\frac{1}{{\left[Y(a)\sqrt{\pi a}\right]}^{m}}da$$ (3)

Assuming that the number of loads experienced by the structure per year is *N*_an_ and the service time is *t*, without considering the increase in annual traffic (i.e., the number of annual stresses acting *N*_an_ is constant), we have *N* = *N*_an_·*t*. Bringing Eq (2) and Eq (3) into Eq (1) and solving by integration, the relationship between the crack size a and the service time t can be obtained as follows:

$$a={\left(a_{0}^{(2-m)/2}+\frac{2-m}{2}CS_{sr}^{m}Y{\left(a\right)}^{m}\pi^{m/2}N_{an}t\right)}^{\frac{2}{2-m}}$$ (4)

## 3 Prediction of lining service life considering crack expansion

### 3.1 Probability of detection

Inspection can identify the cracks in the lining and, as a result of different tests, determine whether the lining needs maintenance and what appropriate repair measures should be taken. Commonly used methods of tunnel inspection include on-site visual observation and non-destructive testing. The factors affecting the accuracy of crack detection are numerous, complex, and uncertain. The testing results can be directly affected by the testing instruments’ accuracy, testing location, and the level of operation of the personnel. Whether damage can be inspected is related to the recognition probability of the inspection methods and the degree of damage development. Several models are available to describe the probability of detection (PoD) function. Among the widely used models is the log-normal PoD function, which can be expressed as [15]:

$$\mathrm{PoD}=0\;\mathrm{for}\;a\leq a_{\min}$$ (5A)

$$\mathrm{PoD}=\Phi [\frac{\ln(a)-\ln(\alpha )}{\beta }]\;\mathrm{for}\;a>a_{\min}$$ (5B)

Where Φ(·) is the standard normal cumulative distribution function (CDF); *a* is the crack depth; *α* is the location parameter indicating the damage value when PoD = 0.5; *β* is the scale parameter; *a*_min_ indicates the minimum crack detection value. *α*, *β* and *a*_min_ are all related to the inspection method.

### 3.2 Fatigue life of lining cracks considering PoD and maintenance

**Fig 1:**
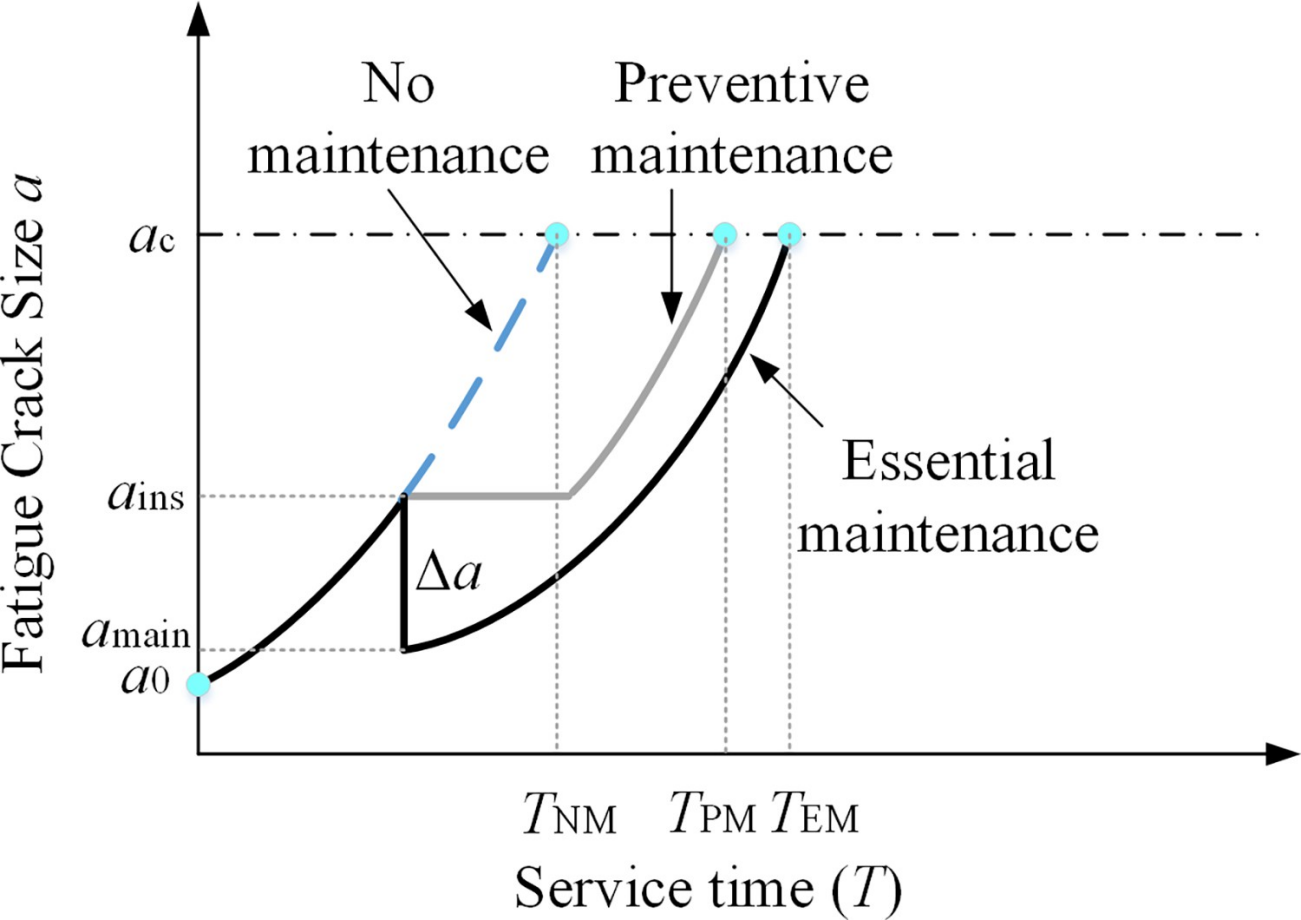
Crack size with three maintenance strategies.

Lining maintenance is essential to ensure the lining structure maintains good durability and safety during its service life. Using different maintenance measures will produce varying maintenance effects and impact the tunnel service life differently. For cracked liners, three strategies are usually used: no maintenance, preventive maintenance, and essential maintenance to ensure the safety performance of the lining structure during the service life of the tunnel. Preventive maintenance refers to measures to improve the service life of the lining by preventing or retarding the expansion of cracks, such as using epoxy filling and coating. Essential maintenance, which can be equated to improving the service life of the lining by reducing the crack size to a shorter state, such as reinforcement by applying steel sheets or fiber reinforced plastic (FRP) and reconstruction. The crack development curves of cracked liners for the three maintenance strategies are given in Fig 1.

In Fig 1, *T*_NM_ indicates the service life when the lining cracks expand from the initial crack depth *a*_0_ to the ultimate crack depth *a*_c_ without maintenance measures; *T*_PM_ indicates the service life after delaying the crack expansion Δ*T*_PM_ when preventive maintenance measures are adopted; *T*_EM_ indicates the service life after reducing the crack size Δ*a* when essential maintenance measures are adopted.

After preventive maintenance or essential maintenance, the service life of the tunnel lining can be expressed as:

$$T_{M}=\{\begin{array}{ll} T_{NM}+\Delta T_{PM}=T_{N}+5k_{1} & \mathrm{Preventive}\;\mathrm{maintenance}\\ T_{NM}+\Delta T_{EM}=T_{N}+15k_{2} & \mathrm{Essential}\;\mathrm{maintenance} \end{array}$$ (6)

Where Δ*T*_PM_ and Δ*T*_EM_ indicate the growth value of lining life after preventive maintenance and necessary maintenance, respectively; *k*_1_ and *k*_2_ indicate the maintenance effect of preventative maintenance and essential maintenance, respectively, 0.1 ≤ *k* ≤ 0.9, the larger the *k* value indicates the better the maintenance effect.

Further allowing for the uncertainty of crack detection and maintenance measures, the fatigue service life of the tunnel lining fatigue cracks after detection and maintenance is obtained as:

$$T_{\mathrm{life},\mathrm{main}}=(1-\mathrm{PoD})\cdot T_{NM}+\mathrm{PoD}\cdot T_{M}$$ (7)

## 4 Optimization of lining cracking maintenance strategy

### 4.1 Lining inspection and maintenance costs

In the tunnel service process, as the lining cracks continue to expand, timely inspection and maintenance measures can effectively improve the performance of the tunnel lining. But at the same time, the cost of expenditure also increases accordingly. Therefore, in the actual inspection and maintenance work, it is necessary to consider the inspection and maintenance effect and the cost of the inspection and maintenance measures taken. For the fatigue crack of tunnel lining, the whole life cycle cost is divided into inspection cost *C*_ins_, maintenance cost *C*_main_ two parts. In this paper, only the ultrasonic detection method is considered, and its inspection cost *C*_ins_ is considered a constant.

Generally, it is considered that the appropriate maintenance measures are taken immediately after the crack inspection is performed. The cost of maintenance of cracks is related to the maintenance effect, and it is typically regarded that the better the maintenance effect is, the higher the maintenance cost is. Considering the uncertainty of lining crack detection, the maintenance cost *C*_main_ of lining fatigue crack is taken as [15]:

$$C_{\mathrm{main}}=\mathrm{PoD}\cdot C_{M}{\left(1-0.7k\right)}^{r_{m}}$$ (8)

Where *C*_M_ is a constant, RMB, *r*_m_ is a positive integer, and both *C*_M_ and *r*_m_ are related to maintenance measures.

In summary, the total cost *C*_total_ for fatigue crack inspection and maintenance of the tunnel lining over its full life cycle can be expressed as:)

$$C_{total}=C_{ins}+C_{main}$$ (9)

### 4.2 Multi-objective optimization of maintenance strategies

Tunnel maintenance decisions can be abstracted as a mathematical optimization problem. Various combinations of detection methods and maintenance measures for fatigue crack expansion in tunnel linings correspond to different lining fatigue life expectations and detection and maintenance costs. The maintenance strategy optimization aims to select a reasonable inspection time, the best inspection method, and the optimal maintenance measures based on the inspection results, thus ensuring the tunnel lining’s safety and low maintenance cost within its specified service life.

As mentioned earlier, a bi-objective optimization process is proposed in this paper. With the objectives of maximizing the lining service life and minimizing the inspection and maintenance costs, the maintenance strategy for lining with fatigue cracks is optimized. The mathematical formulation of the above optimization problem is:

$$\begin{array}{l} \overset{\mathrm{minmize}}{\mathrm{PM},\mathrm{EM}}\{C_{\mathrm{total}}\}\\ \overset{\mathrm{maxmize}}{\mathrm{PM},\mathrm{EM}}\{T_{\mathrm{life},\;\mathrm{main}}\}\\ \mathrm{where}\;\mathrm{PM}=\{T_{ins},K\}\\ \;\mathrm{EM}=\{T_{ins},K\} \end{array}$$ (10)

$$T_{ins}=(t_{ins,1},\cdots ,t_{ins,i},\cdots t_{ins,n})$$ (11)

$$K=(k_{1},\cdots ,k_{i},\cdots k_{n})$$ (12)

Where PM and EM correspond to preventive maintenance and necessity maintenance in Fig 1, respectively; $\overset{\mathrm{minmize}}{\mathrm{PM},\mathrm{EM}}\{C_{\mathrm{total}}\}$ is the optimization problem of the minimum value of the objective function *C*_total_ under the two maintenance strategies; $\overset{\mathrm{maxmize}}{\mathrm{PM},\mathrm{EM}}\{T_{\mathrm{life,main}}\}$ is the optimization problem of the maximum value of the objective function *T*_life,main_ under the two maintenance strategies; the design variables are the inspection time vector ***T***_**ins**_ and the maintenance effect vector ***K***, where *t*_*ins*,*i*_ denotes the *i*th detection time, and *k*_*i*_ represents the *i*th maintenance effect parameter.

### 5 Case study

**Fig 2:**
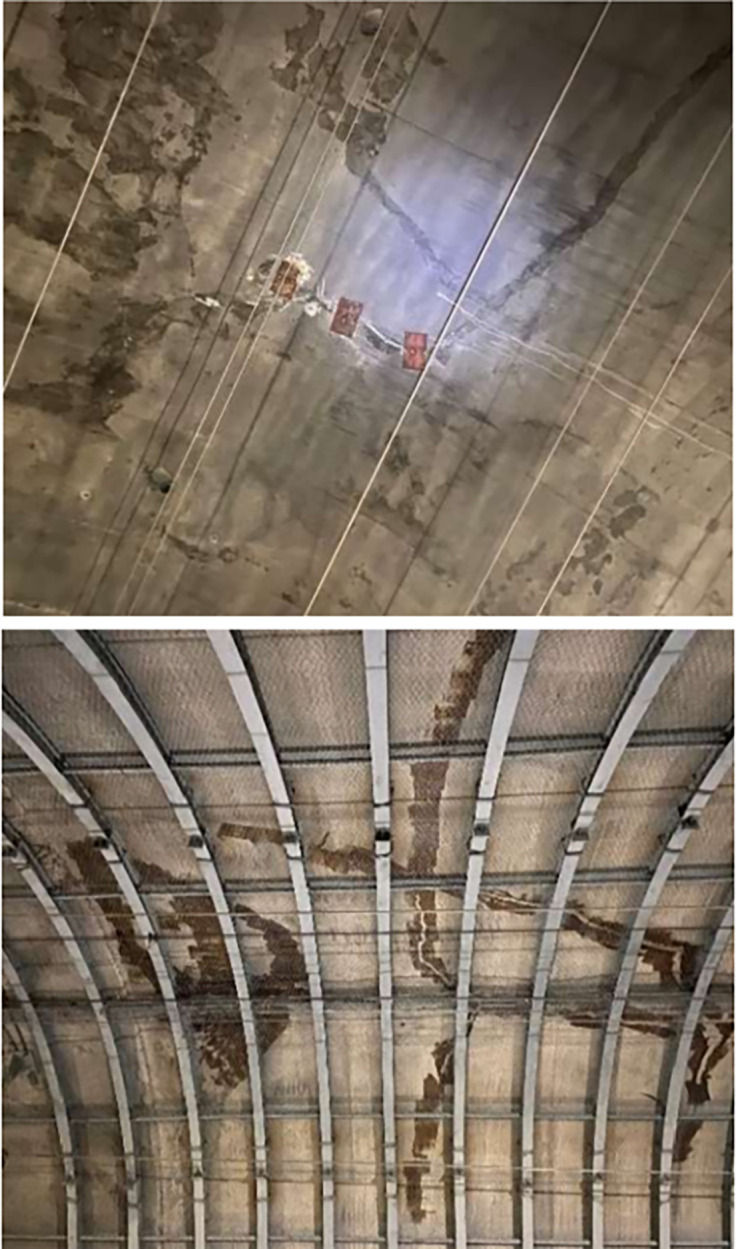
Tunnel lining cracks: (a) arch cracking; (b) sidewall cracking.

**Fig 3:**
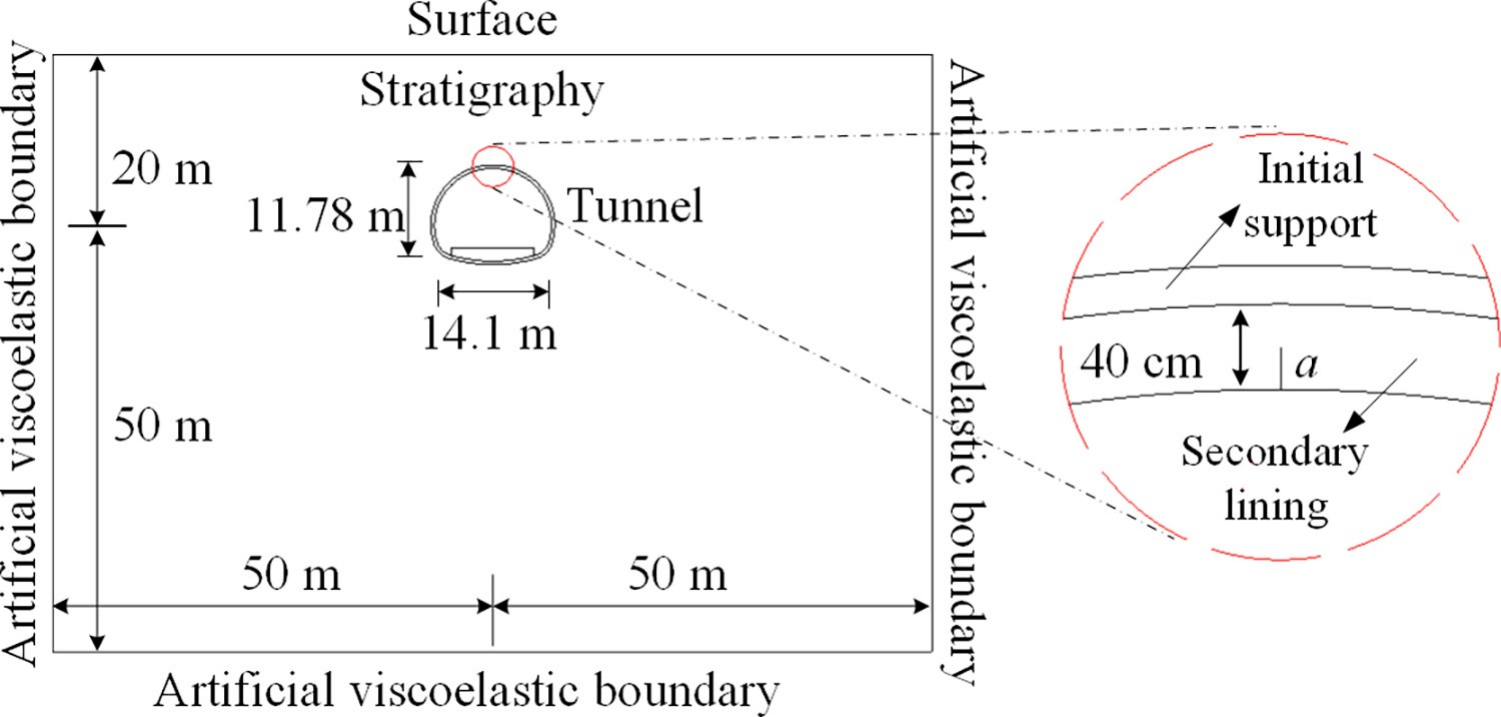
Dimensions of the calculated model.

A two-lane railroad tunnel has been in operation for more than 10 years, with a total length of 3769 m and a maximum depth of about 290 m. The lining type is the composite lining, and the thickness of the secondary lining is 40 cm. The lining is detected to have several longitudinal cracks distributed in different perimeter rock levels and manifested by longitudinal cracking in the tunnel arch and sidewalls, as shown in Fig 2. The crack expansion problem was simplified to a two-dimensional plane strain model for simulation to study the fatigue expansion of lining cracks under dynamic train load. To facilitate the analysis, the coupled dynamic model in which the soil, lining, and filling layers are simplified to an isotropic, homogeneous elastic medium. The mechanical parameters of the surrounding rock and concrete are shown in Table 1. The numerical model dimensions are shown in Fig 3.

**Table 1:** Material parameters.

Material	Grade	Density/(kg·m^-3^)	Elastic modulus/GPa	Poisson’s ratio
Surrounding rock	V	1800	1.5	0.4
Initial support	C25	2300	30	0.2
Secondary lining	C35	2650	32.5	0.2
Filling layer	C25	2300	30	0.2

In the direct dynamic analysis, the reflection effect of the boundary on the vibration load is eliminated by setting viscoelastic boundary at the bottom and left and right sides of the model [31]. The viscoelastic boundary parameters are calculated as:

$$K_{\mathrm{BN}}=\frac{1}{1+A}\cdot \frac{\lambda +2G}{2r},C_{\mathrm{BN}}=B\rho C_{P}$$ (13)

$$K_{\mathrm{BT}}=\frac{1}{1+A}\cdot \frac{G}{2r},C_{\mathrm{BT}}=B\rho C_{S}$$ (14)

where *K*_BN_ and *K*_BT_ denote the normal and tangential spring stiffness, respectively; *C*_BN_ and *C*_BT_ denote the normal and tangential damping coefficients, respectively; *λ* and *G* are lame constants; *ρ* is the density of the medium; $C_{P}=\sqrt{\frac{\lambda +2G}{\rho }}$ and $C_{S}=\sqrt{\frac{G}{\rho }}$ denotes the P-wave and S-wave wave speeds, respectively; *r* is the distance from the geometrical center to the artificial boundary; and *A* and *B* are the adjusting coefficients, which are taken to be *A* = 0.8 and *B* = 1.1.

**Fig 4:**
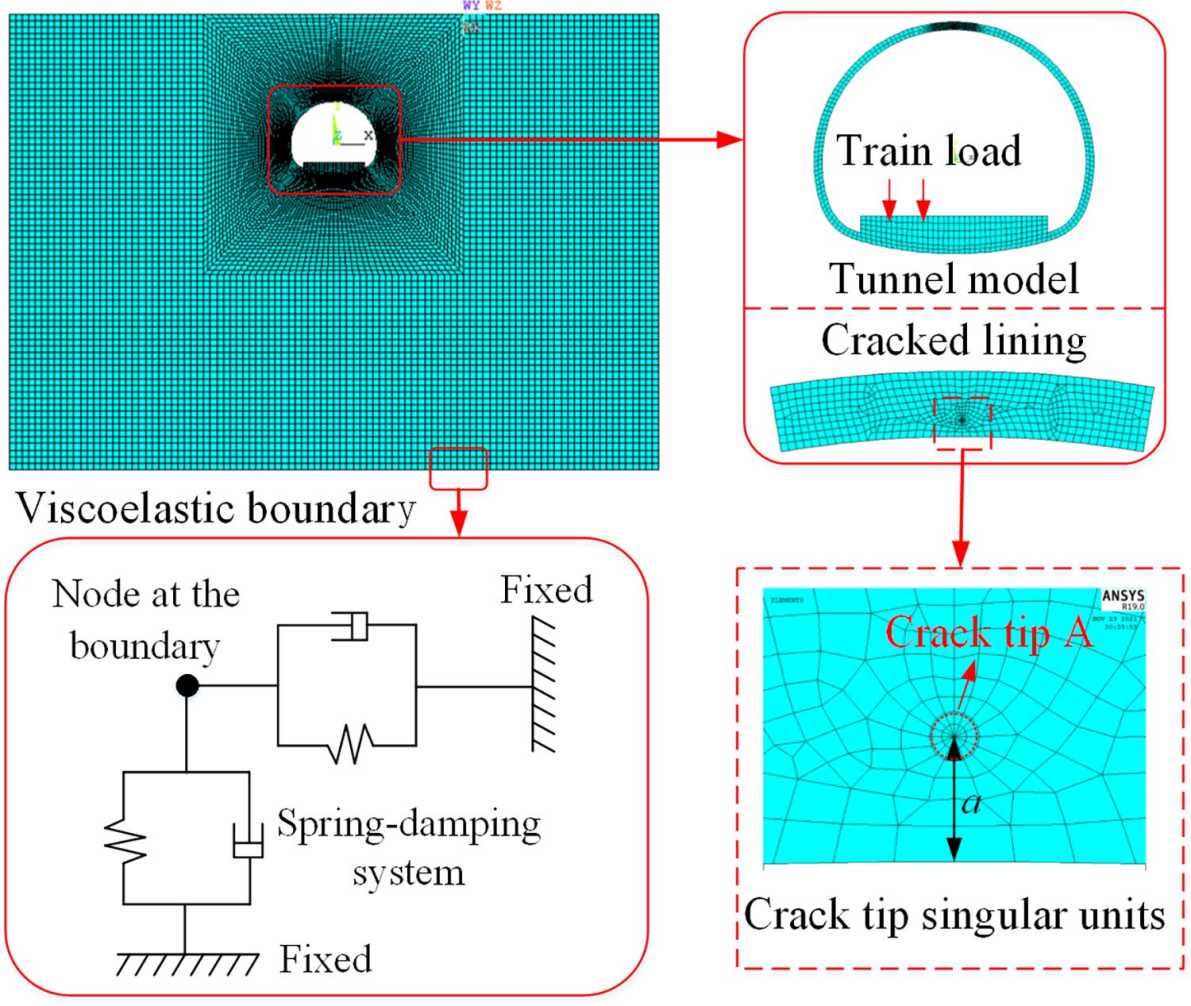
Cracked tunnel-strata coupled finite element model.

A numerical model of the crack-containing tunnel-stratum coupling was established using ANSYS software, as shown in Fig 4.

The Rayleigh damping is used for the computational model, and its expression is given as follows:

[C]=α[M]+β[K] (15)

Where *α* and *β* are the mass damping and stiffness damping coefficients, respectively; [*M*] and [*K*] are the structural mass matrix and stiffness matrix, respectively. *α* and *β* can be calculated from the structural vibration frequency and vibration damping ratio:

$$\left\{\begin{array}{l} \alpha \\ \beta \end{array}\}=2\frac{\omega_{i}\omega_{j}}{\omega_{i}^{2}-\omega_{j}^{2}}[\begin{array}{c} \omega_{i}\;-\omega_{j}\\ \frac{-1}{\omega_{i}}\;\frac{1}{\omega_{j}} \end{array}]\{\begin{array}{l} \xi_{j}\\ \xi_{i} \end{array}\right\}$$ (16)

Where *ω*_*i*_ and *ω*_*j*_ are the ith and jth order frequencies that contribute to the structural vibration; *ξ*_*i*_ and *ξ*_*j*_ are the damping ratio of the ith and jth order vibration modes, respectively. The first two order frequencies of the modal analyzed model are 3.238 5 Hz and 4.792 8 Hz, the damping ratio is taken as 5%, and the damping coefficients *α* = 0.1933 and *β* = 0.0125 are obtained by calculation.

### 5.1 Train loads

**Fig 5:**
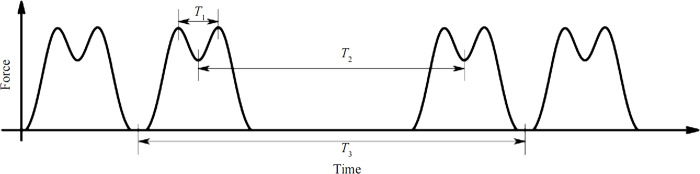
Schematic diagrams of M-shaped wave.

Reasonable selection of train vibration loads is the focus of lining crack expansion studies. The test results show that the train load is a complex issue involving many factors such as train axle weight, suspension system, travel speed, track composition, line smoothness, etc., simultaneously, and the train load subjected to the structure is a one-way impulse stress wave. The loads obtained from the actual tests differ significantly from the uniform load pattern. While converting the impulse stress into a uniform load is a simple way to calculate the deformation based on static calculation, it is undesirable when considering dynamic effects. For high-speed or heavy railroads, due to the increase in speed and train density, the structure is subjected to increased loads and an increased number of actions. It is necessary to consider the dynamic effects of the train operation and its repeated fatigue effects on the structure. The vibrating load on the tunnel lining is mainly generated by the train motion, which primarily depends on the vehicle mass, running speed, and wheelbase. Assuming that the carriage weight is concentrated on the four-wheel sets, one vibration wave is generated when one wheel set moves one position, and another wave is generated when another wheel set moves that position on the same bogie. An m-shaped wave of the train load was given by Niu et al. [32], as shown in Fig 5. *T*_1_ indicates the time when two adjacent wheel sets of the same bogie pass through a position; *T*_2_ indicates the time when two bogies of the same carriage pass through a position; and *T*_3_ indicates the time when the carriage passes through a position.

The high-speed trains in the Beijing-Shanghai high-speed railroad operation can be divided into four types: CRH380A/B and CRH380AL/BL. A and B distinguish chiefly by the allocation of drive motors. The " L " means that the train has 16 cars. Otherwise, it is 8. The side view of the CRH380BL configuration is shown in Fig 6 from Feng et al. [33].

**Fig 6:**
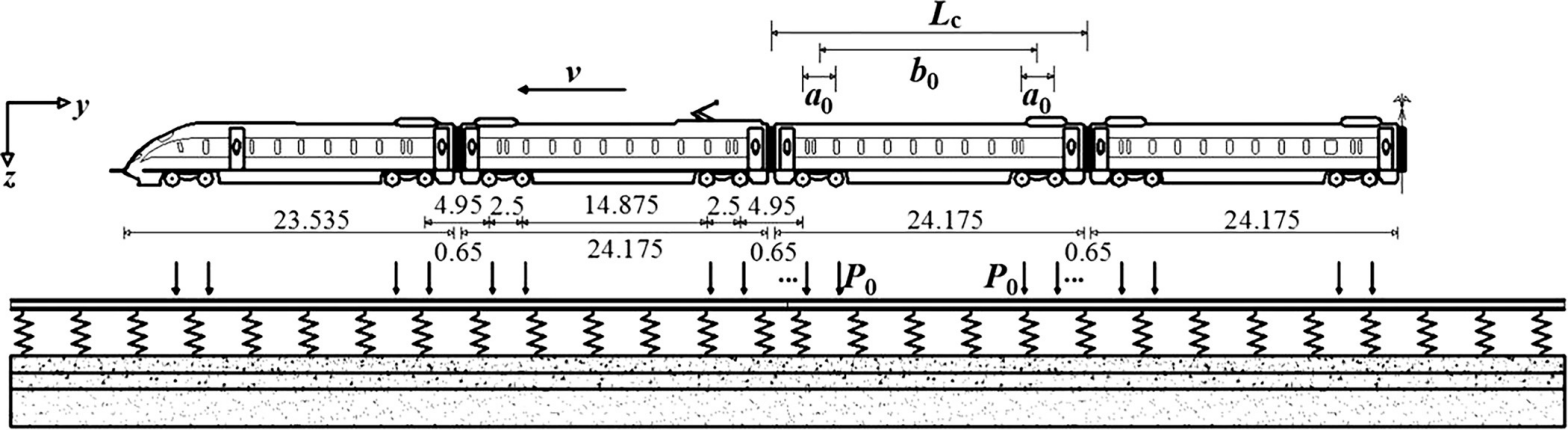
Configuration of the CRH3 train used in China.

Bian [34] studied the transfer law of train load by model test and obtained the load-sharing ratio of fasteners under the action of wheel-rail load. Further, the time-varying curve equation of vibration load on the pins on the track under the movement of the trainload of the formation was obtained:

$$f(v,t)=\{\begin{array}{l} P_{0}\gamma (vt-x_{0})\;\mathrm{Single}\;\mathrm{wheel}\;\mathrm{pair}\\ \sum_{i=1}^{2}P_{0}\gamma (vt-x_{i})\;\mathrm{Single}\;\mathrm{bogie}\\ \sum_{i=1}^{n}\sum_{j=1}^{4}P_{0}\gamma (vt-x_{ij})\;n\;\mathrm{carriages} \end{array}$$ (17)

$$\gamma (x)=\alpha \;\exp(-\frac{x^{2}}{2\omega^{2}})$$ (18)

Where *P*_0_ is the wheel track vertical force, taking 70 kN; *x*_*ij*_ is the train axle position; v is the train speed; *t* is the train running time; *ω* is a constant. Corresponding to the longitudinal influence range under the action of a single axle load, taken as 0.78; *α* is a constant, corresponding to the maximum load ratio of fasteners under the cut of a single axle load, taken as 0.34.

**Fig 7:**
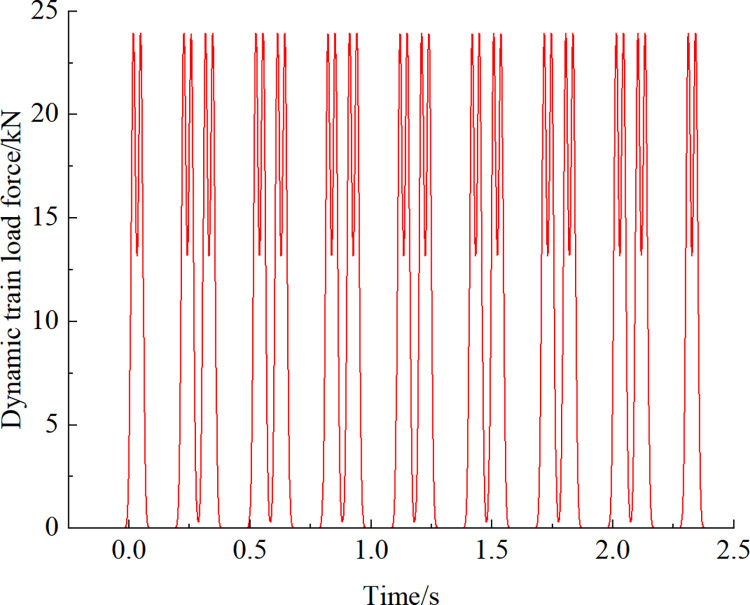
Calculated train loads.

When the train runs at speed equal to 300 km/h (the maximum operating speed of CRH380A/B), the corresponding dynamic train load is shown in Fig 7. The maximum value of the train load is about 23.94 kN, and the time of train passage is about 2.5 s. Subsequently, the total number of analysis steps is 2500, where the interval is 0.001 s.

### 5.2 Analysis of the results

**Fig 8:**
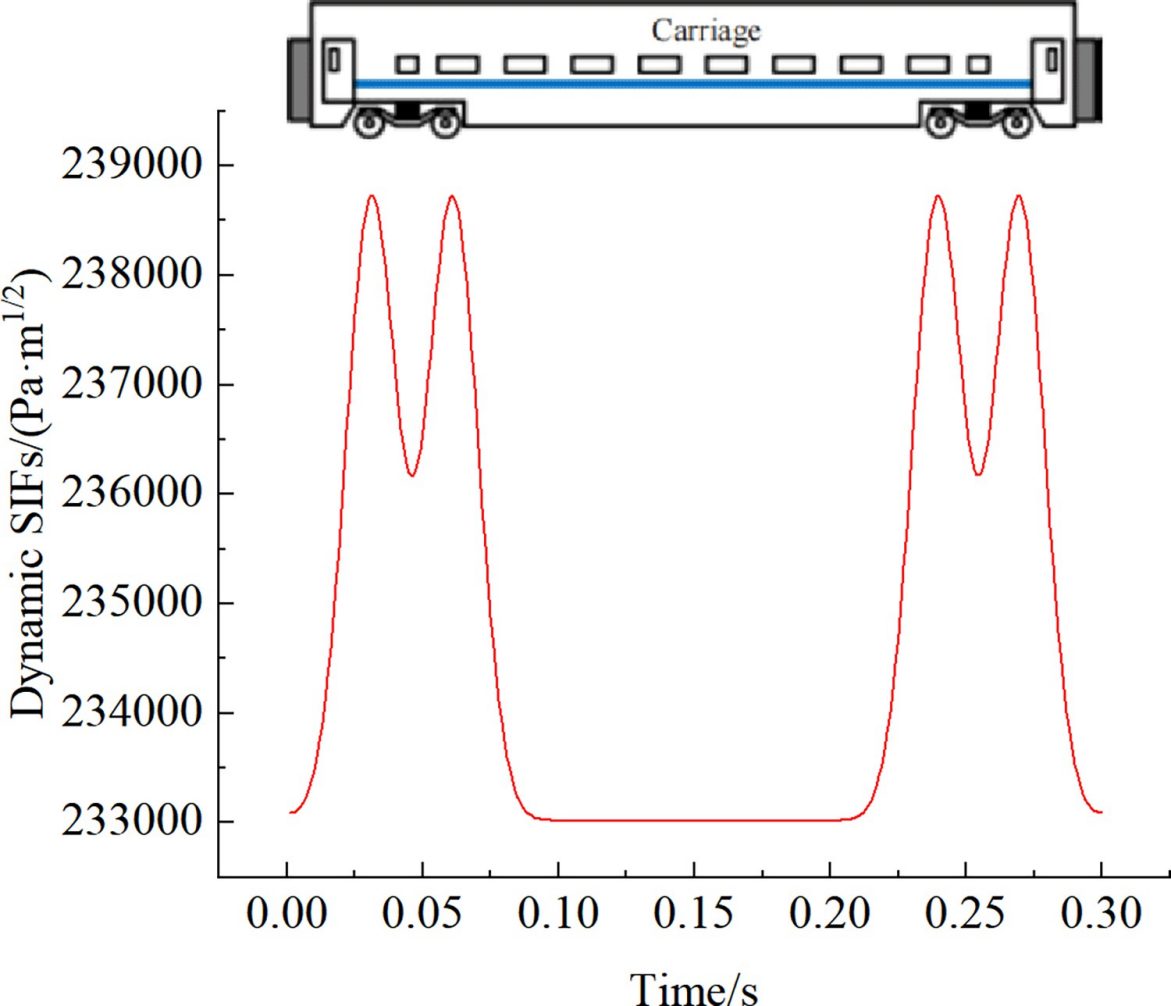
Time course curve of SIFs.

The strength of the stress field at the crack tip is an essential factor in determining the stability or expansion of the crack. Stress intensity factors (SIFs) are mathematically defined to solve the singularities of the stress and strain fields near the crack tip. The SIFs for different loading steps were calculated using the 1/4 node displacement extrapolation method [14] using the fracture module provided by ANSYS software. According to the field survey results, the depth of liner cracks is widely distributed. To study the variation of training power SIF amplitude and crack depth, the crack depths of coupled power models were 50 mm, 75 mm, 100 mm, 125 mm, 150 mm, and 175 mm. Whose normalized depth with respect to tunnel lining thickness *a*/*h* was 0.1250, 0.1875, 0.2500, 0.3125, 0.375, and 0.4375 respectively. Taking the 15 cm crack at the vault top as an example, the time course of the dynamic SIFs of the lining crack when a single train passes through the calculated section is shown in Fig 8. It can be seen that the waveform of the dynamic SIFs is similar to the train load, with the maximum value *K*_max_ = 238734Pa·m^1/2^, the minimum value *K*_min_ = 233017Pa·m^1/2^, and the maximum magnitude *ΔK* = 5717Pa·m^1/2^. Under the action of train vibration load, the SIFs at the crack tip will increase, leading to crack opening and further crack expansion.

**Fig 9:**
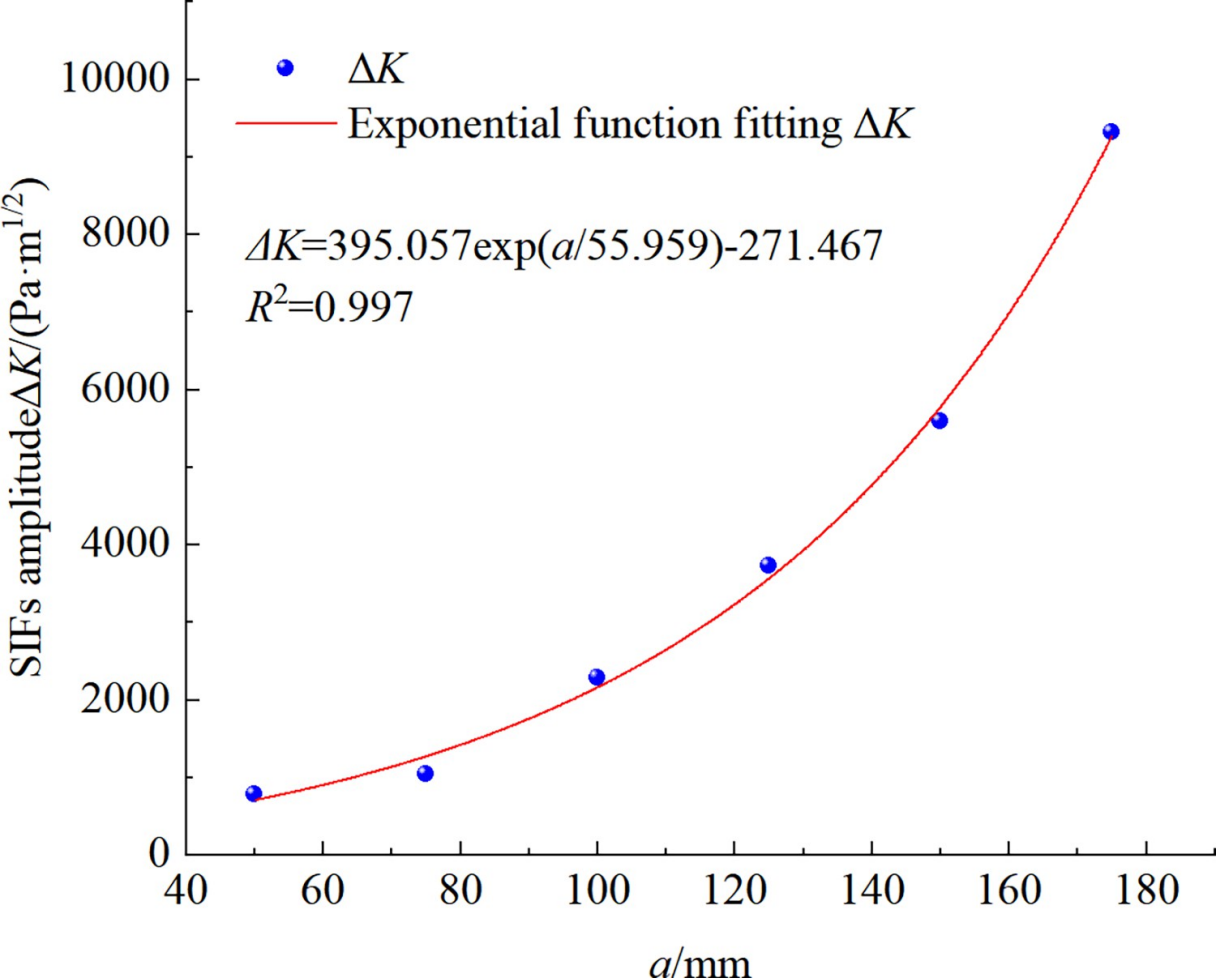
SIFs amplitude of cracks in lining at different depths.

According to the calculated results, the variation of the stress intensity factor amplitude Δ*K* with the crack depth a is shown in Fig 9. As can be seen from the figure, the relationship between the stress intensity factor amplitude Δ*K* and the crack depth approximately satisfies the exponential function. The amplitude of the stress intensity factor increases with increasing crack depth and grows at an increasing rate. It shows that the rate of fracture extension increases with the extension of fracture depth, which is consistent with the results of Virkler [35].

After investigation, the design calculation working condition is 120 pairs of passing trains per day, the ratio of 16 train formations and 8 train formations is about 3:1, and each formation can be considered to be repeatedly loaded 4 times. Then the annual number of train actions on the lining structure is *N*_an_ = (8 × 30 + 16 × 90) × 4 × 365 = 2452800 times, and the remaining parameters are taken as shown in Table 2.

**Table 2:** Parameter values.

Parameter	*a*_0_/(mm)	*C* [36]	*m* [36]	*N*_*an*_/(times·a^-1^)
**Value**	0.1	1.041 8×10^−9^	3.131 6	2 452 800

**Fig 10:**
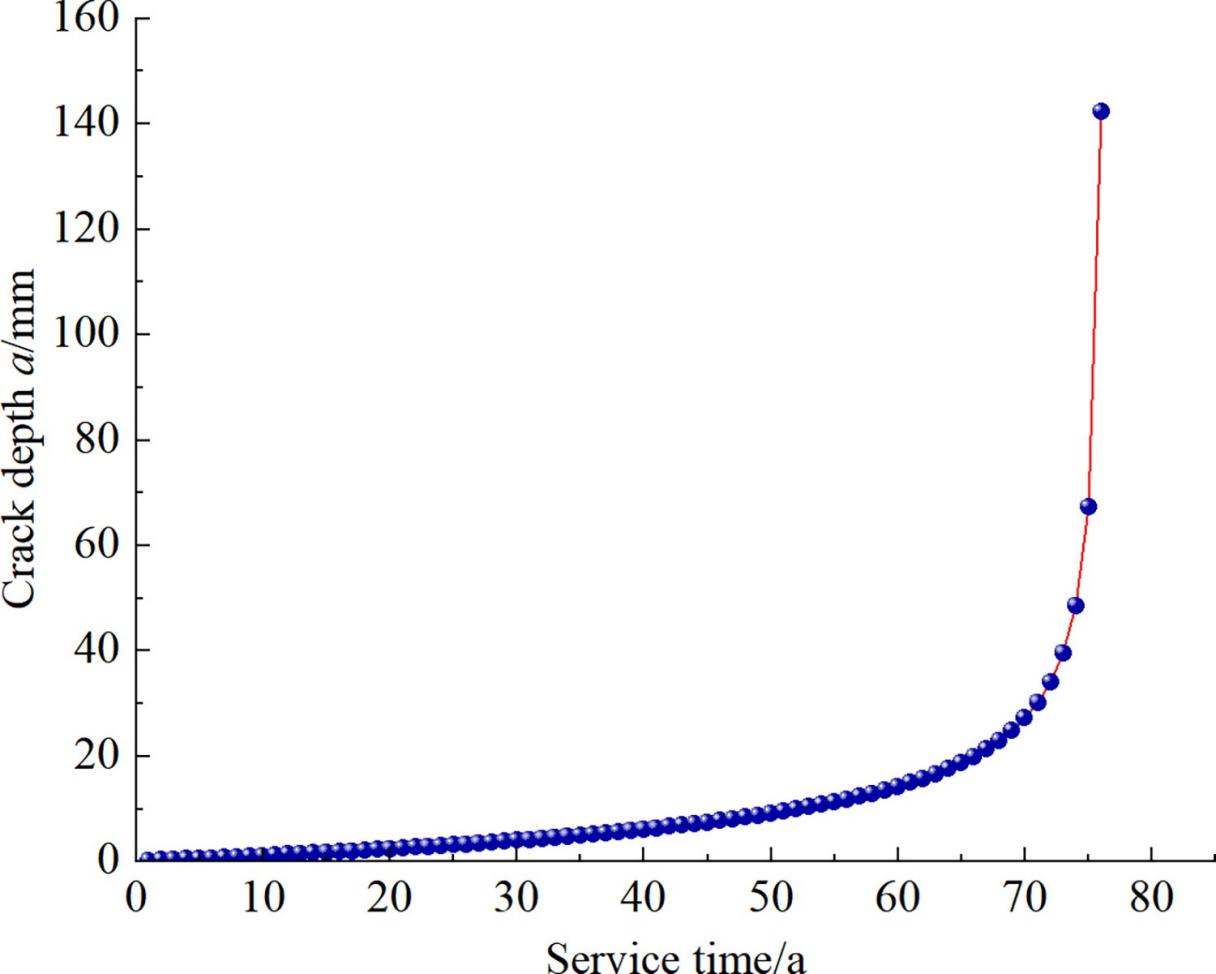
Crack expansion trajectory.

Assuming the existence of 0.1mm cracks at the beginning of tunnel service, the curve of crack depth versus service time is obtained according to the SIFs amplitude expression in Fig 9, as shown in Fig 10. When the tunnel service time is less than 40 years, the crack growth rate is slow, and after more than 40 years, the crack growth rate is significantly accelerated. Taking the ultimate crack depth of lining *a*_c_ = 150 mm, the crack expansion reaches the limit value at 76 years of service, which does not meet the life requirement of 100 years.

### 5.3 Maintenance strategy optimization

**Fig 11:**
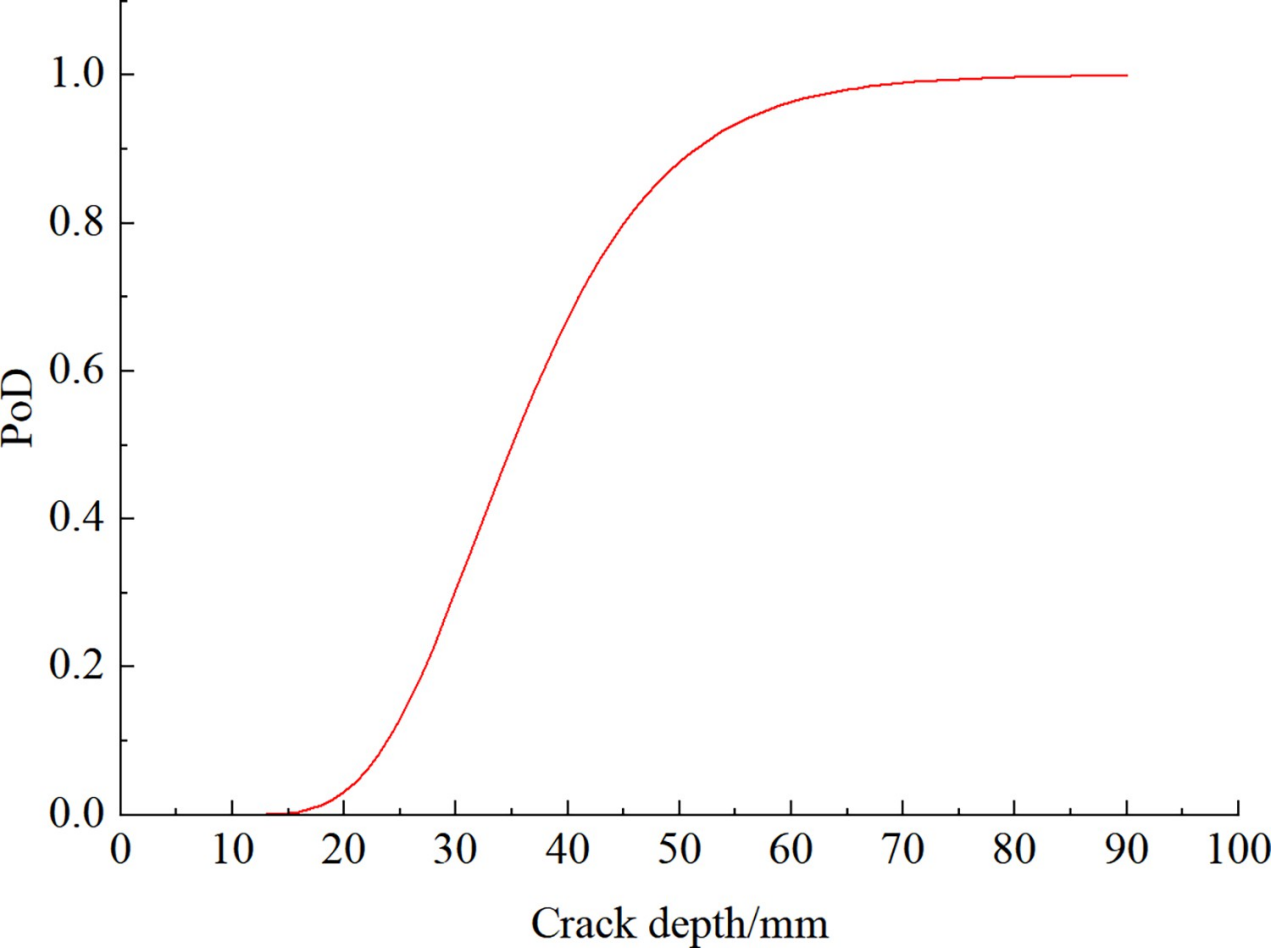
PoD of cracks by ultrasonic method.

The lining cracks were detected by ultrasonic detection method, and the probability of detection (PoD) was calculated according to Eq (5), taking *α* = 35 mm, *β* = 0.3; *a*_min_ = 10 mm. the PoD at different crack depths is shown in Fig 11.

The lining cracks were detected by ultrasonic detection method. They are specifying the lining crack depth threshold as *a*_c_ = 150 mm, the cost parameters of crack detection *C*_ins_ = 2 000 RMB, *C*_PM_ = 50 000 RMB, *C*_EM_ = 80 000 RMB, *r*_m_ = 4. The NSGA-II genetic algorithm toolbox provided by MATLAB version R2014b is used for optimization calculations and using the Pareto optimization frontier to determine the critical parameters of the structural maintenance strategy. The maximum number of generations is fixed at 200 with population of 100. The design variable constraints are:

$$\left\{ \begin{array}{l} t_{ins,l}-t_{ins,l-1}\geq 1\\ 0.1\leq k\leq 0.9 \end{array}\right.$$ (19)

That is, the interval between 2 adjacent inspections is greater than or equal to 1 year, and the maintenance effect is between 0.1 and 0.9.

**Fig 12:**
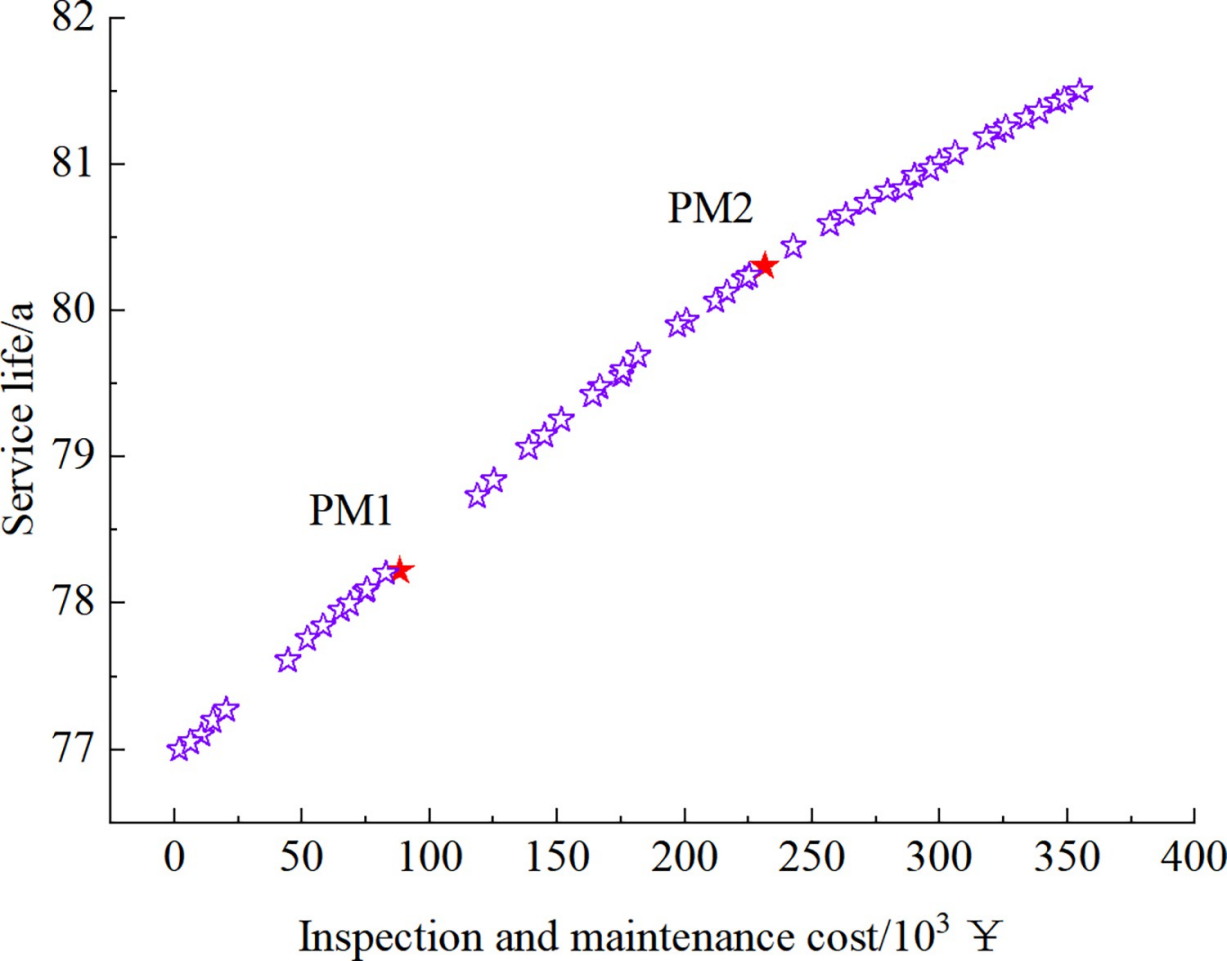
Pareto-optimized solution set under preventive maintenance strategy at 1-inspection maintenance.

**Fig 13:**
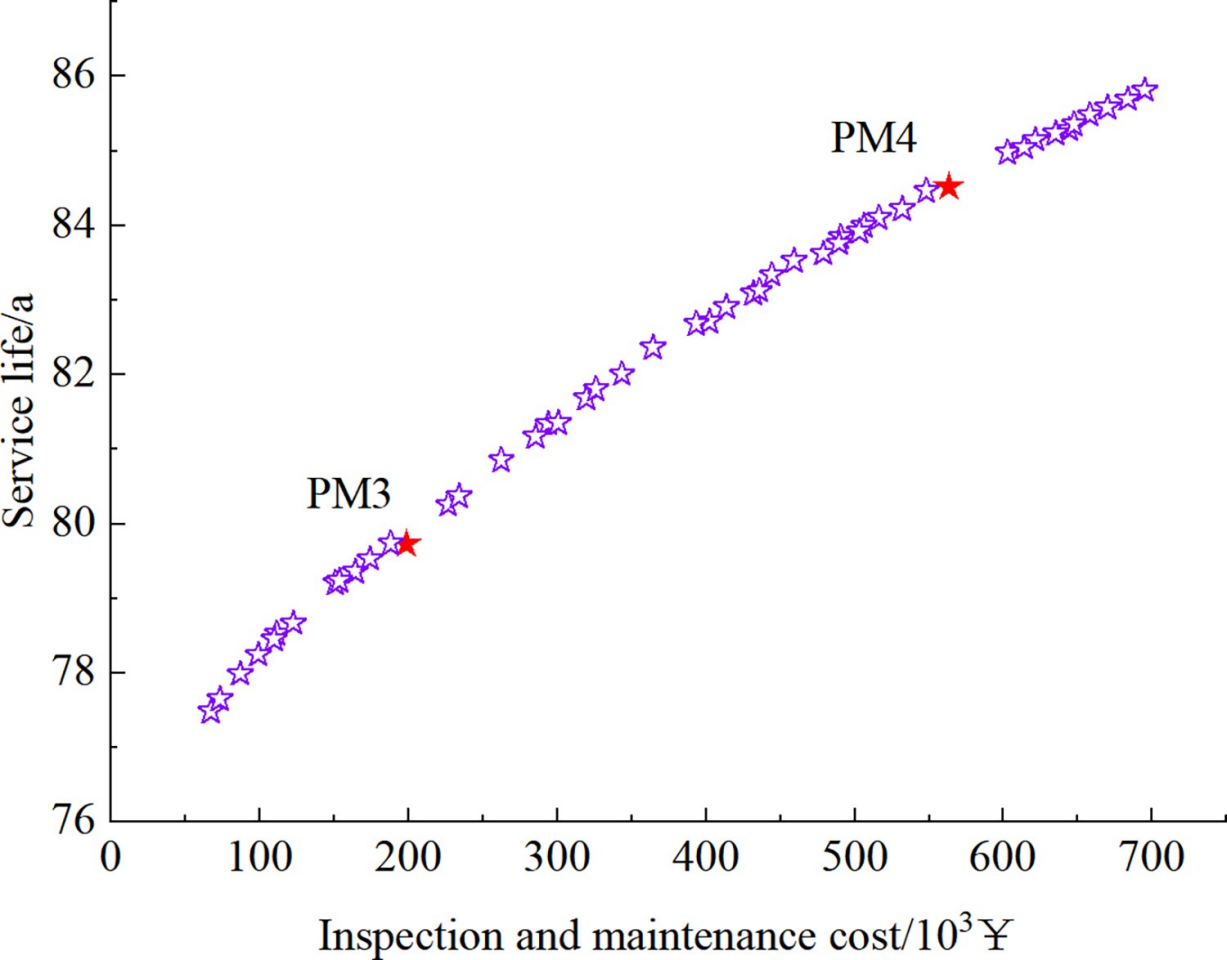
Pareto-optimized solution set under preventive maintenance strategy at 2-inspection maintenance.

**Fig 14:**
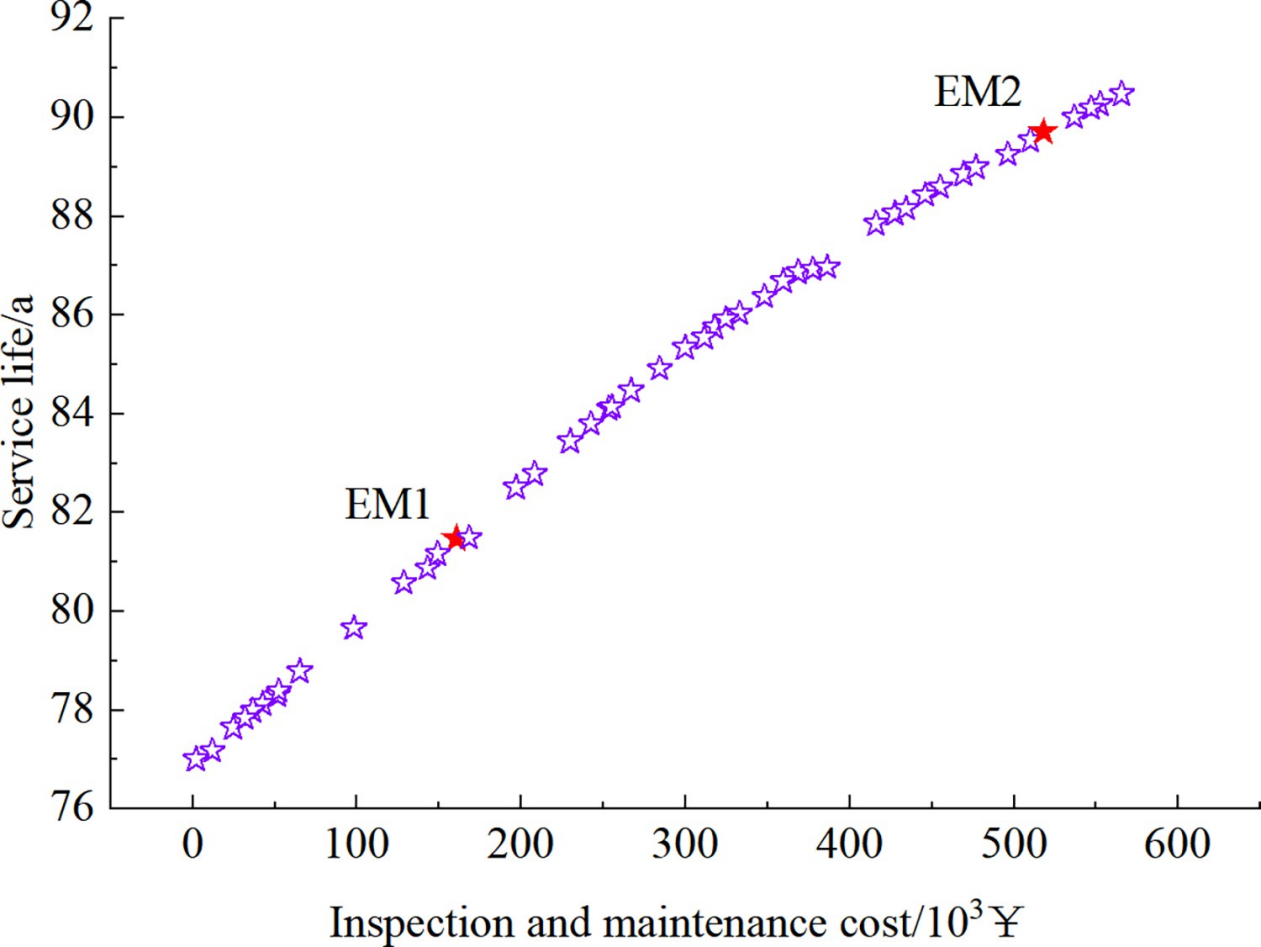
Pareto-optimized solution set under essential maintenance strategy at 1-inspection maintenance.

**Fig 15:**
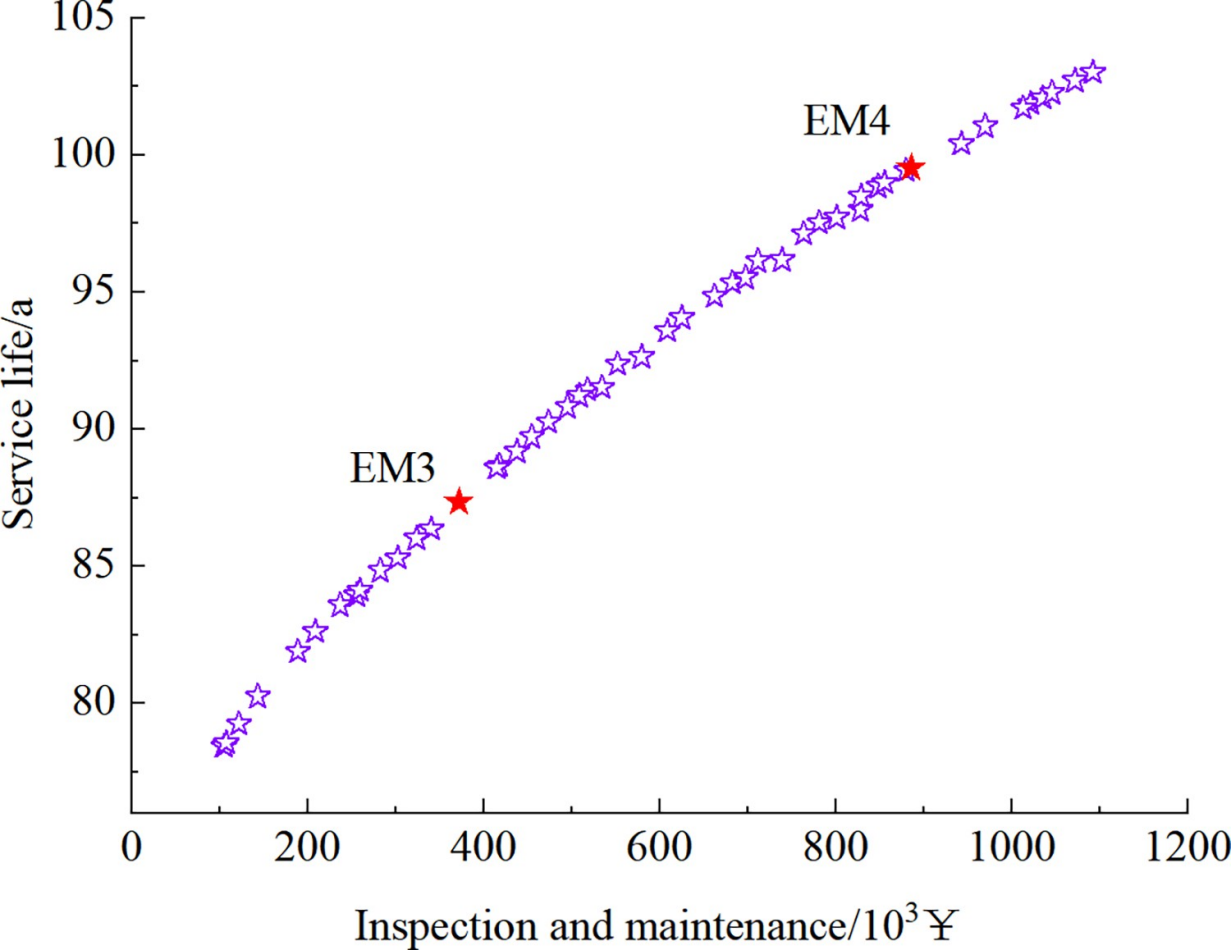
Pareto-optimized solution set under essential maintenance strategy at 2-inspection maintenance.

Figs 12–15 show the set of Pareto-optimized solutions for the tunnel vault fatigue crack detection and maintenance strategy calculated after using preventive maintenance and necessity maintenance for one and two times, respectively. Each of these points corresponds to an optimization strategy for lining life and cost, which contains information on the crack inspection time and the results that maintenance measures can achieve.

The strategies EM1~EM4 and PM1~PM4 in Figs 12 to 15 are selected as 8 representative solutions (8 optimized maintenance strategies). The adopted inspection and maintenance time, maintenance measures, corresponding fatigue life, and total cost of inspection and maintenance are shown in Table 3.

**Table 3:** Calculated results of design variables and objective functions.

Pareto optimum solution	Number of inspections (*n*)	Expected life/years	Total Cost/10^3^ RMB	Inspection time		Maintenance Effect	
				*t*_ins,1_	*t*_ins,2_	*k*_1_	*k*_2_
PM1	1	68.22	88.33	62.1	-	0.438	-
PM2	1	80.30	231.33	75.1	-	0.760	-
PM3	2	79.73	198.50	73.5	76.5	0.254	0.351
PM4	2	84.52	563.28	74.5	78.9	0.643	0.896
EM1	1	81.48	160.63	72.8	-	0.500	-
EM2	1	89.71	518.02	75.2	-	0.849	-
EM3	2	87.34	372.01	71.0	84.15	0.584	0.513
EM4	2	99.54	885.30	74.2	87.05	0.770	0.791

As can be seen in Figs 12 to 15 and Table 3, as the cost of inspection and maintenance increases, the expected value of lining service life increases accordingly. However, the increase is relatively lower in the later years of service, indicating that the investment in inspection and maintenance should be increased as the service life of the tunnel increases. Comparing the solutions of PM2 and EM1: when the difference in service life expectancy is insignificant, the cost of essential maintenance is significantly less than that of preventive maintenance, indicating that essential maintenance measures are better. Comparing the solutions of EM2 and EM3: when the difference in service life expectancy is not significant, the cost required for 2 times of inspection and maintenance is significantly lower than that of 1 time. Moreover, the maintenance effect is higher when inspection and maintenance 1 time compared with 2 times inspection and maintenance, indicating that inspection and maintenance 1 time requires higher maintenance quality and standard to ensure the service performance of the structure.

However, it should be noted that, although the calculations show that the essential maintenance and the increase in the number of inspections and maintenance can improve the structure’s service life with a lower cost, it also implies a higher level of management and a more tedious maintenance process. In summary, in the essential maintenance of tunnels, the tunnel management can choose from the Pareto optimization set the optimal inspection and maintenance strategy that meets both serviceability and financial requirements, depending on the funds available and the expected service life of the structure.

## 6 Conclusions

In this study, a framework for estimating crack expansion throughout the life cycle based on train loads was proposed to provide tunnel managers with key decision information for planning secondary lining maintenance activities. The growth process of lining cracks was investigated by dynamic analysis, and a multi-objective optimization algorithm model based on a genetic algorithm for the whole-life maintenance strategy of tunnel lining structures was constructed. The developed model was applied to detect and maintain lining cracks in an operational railroad tunnel. The following are the major findings of this work:

1. A coupled tunnel-rock model with cracks was established for lining crack expansion due to long-term train loading. The stress intensity factors at the tip of the lining cracks under train loading are calculated, and the variation law of the stress intensity factor with the crack size is analyzed. Combined with the Paris formula, the expansion trajectory of the tunnel lining cracks under the train load is obtained.
2. Considering the uncertainties affecting the damage inspection methods and maintenance measures, an inspection, and maintenance cost model is given. With maximizing the expected service life after inspection and maintenance and minimizing the inspection and maintenance cost as the optimization objective function, an optimization analysis method based on a genetic algorithm for the inspection and maintenance strategy of cracked concrete lining structures is proposed.
3. A Pareto-optimized solution for the inspection and maintenance strategy of an existing high-speed railroad tunnel is obtained, providing the most beneficial inspection and maintenance strategy for different structural life expectations and cost budgets. The study results can provide a basis for tunnel management to develop a reasonable and optimized inspection and maintenance plan for tunnel linings with crack extension failure caused by train loads.

This paper proposes a detection and maintenance strategy optimization method for tunnel lining vehicle-caused crack expansion through theoretical analysis and numerical simulation. The conclusions are obtained under vault crack conditions, but the way in this paper is applicable to different locations of cracks and loading conditions. It should be noted that the crack expansion behavior in the tunnel length direction has not been considered in this paper; in addition, the tunnel service environment and the aerodynamic effects generated by trains may also impact the lining cracks. Under the influence of multiple factors, the deterioration mechanism of service lining will be more complex, and the above issues need to be further refined in subsequent studies.

## Supporting information

S1 File (ZIP)
